# Hypergranulation over a meshed split-thickness skin graft, a complication of negative-pressure wound therapy: a case report

**DOI:** 10.1186/s13256-022-03521-5

**Published:** 2022-08-30

**Authors:** Masato Shiba, Tomoaki Doi, Hideshi Okada, Ryo Kamidani, Genki Yoshimura, Keigo Kusuzawa, Fuminori Yamaji, Tomotaka Miura, Hideaki Oiwa, Yosuke Mizuno, Ryu Yasuda, Tetsuya Fukuta, Yuichiro Kitagawa, Takahito Miyake, Takahiro Yoshida, Shozo Yoshida, Shinji Ogura

**Affiliations:** 1 Department of Emergency and Disaster Medicine, Gifu University Graduate School of Medicine, 1-1 Yanagido, Gifu, 501-1194 Japan; 2 Department of Abuse Prevention Emergency Medicine, Gifu University Graduate School of Medicine, 1-1 Yanagido, Gifu, 501-1194 Japan

**Keywords:** Debridement, Mesh, Negative-pressure wound therapy, Hypergranulation, Split-thickness skin graft

## Abstract

**Background:**

We present a case of a rare complication of negative-pressure wound therapy (NPWT) wherein there was fixation of a meshed split-thickness skin graft (STSG), suspected as a failure by hypergranulation. However, the meshed STSG was integrated within 5 days of NPWT cessation.

**Case presentation:**

A 22-year-old Asian man sustained 25% total-body-surface-area flame burns. After multiple operations, an ulcer was present on the proximal left thigh. On day 37 after admission, the ulcer was debrided, and an 11/1000-inch (0.28 mm) skin graft was taken from the ipsilateral thigh and meshed, using a 1:1.5 ratio. NPWT was applied to the donor and recipient sites with a continuous negative pressure of 125 mmHg. On day 43, NPWT was discontinued. The skin grafts were not identified on the surface of the granulation tissue. With topical ointment therapy, rapid epithelialization of the ulcer was observed as the granulation tissue regressed. On day 48, the recipient site had completely epithelialized.

**Conclusions:**

The hypergranulation tissue rarely covered the meshed STSGs when the grafts were fixed by NPWT. In that case, immediate debridement should be avoided, and conservative treatment should be initiated.

## Background

Several studies have reported the use of negative-pressure wound therapy (NPWT) for the fixation of split-thickness skin grafts (STSGs) [1, 2] and its complications [3]. Although granulation tissue forms rapidly on wounds treated with NPWT [4], the overgrowth of the granulation tissue is not favorable when the meshed STSG is fixed by NPWT. We present a case of a rare complication of NPWT wherein fixation of the meshed STSG was suspected as a failure by hypergranulation. However, the meshed STSG was integrated within 5 days of NPWT cessation. To the best of our knowledge, this is the first case report of the meshed STSG, which was fully covered by the granulation tissue induced by NPWT.

## Case presentation

**Fig. 1:**
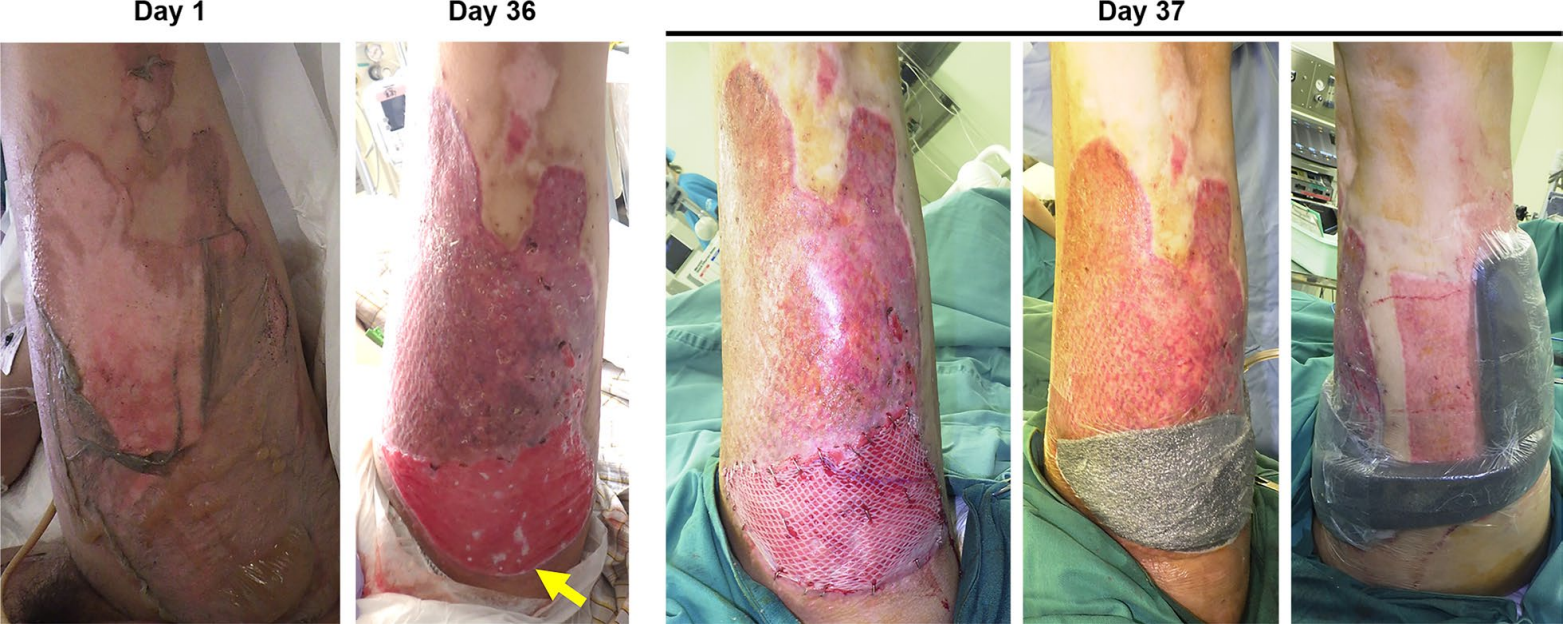
Pre- and intraoperative states of the left thigh. Day 1: the patient’s left thigh with a burn. Day 36: the proximal graft failed, resulting in an ulcer (arrow). Day 37: intraoperative appearance of the skin graft and negative-pressure wound therapy (NPWT). The meshed split-thickness skin graft (STSG) was fixed, and NPWT was applied to both the donor and recipient sites

A healthy 22-year-old Asian male with no specific past medical, social, family, or environmental history presented with extensive burns. In addition, there was no specific medication, smoking history, or alcohol consumption. The cause of the accident was a methanol-based paint spill on his clothes that ignited; this incident occurred at a plating factory (Fig. 1: day 1). He was transferred to our hospital for emergency treatment. On arrival, his vital signs included a respiratory rate of 24 breaths/minute, blood pressure of 165/102 mmHg, and pulse rate of 90/minute. His body temperature was 37.3 degrees Celsius. His Glasgow Coma Scale score was E4 V5 M6, and there was no motor or sensory paralysis. Physical examination revealed 25% total-body-surface-area flame burns (7% partial thickness on the trunk and right hand and 18% full thickness on the right arm, both legs, and chest). There were no problems with hand function. On day 2 after admission, the wounds on his two thighs and right arm were debrided, and Integra dermal regeneration templates (Integra LifeSciences Holdings Corporation, Princeton, NJ, USA) were applied. On day 9 after admission, the wounds on his right lower leg, right arm, and chest were debrided, and 1:3 meshed 10/1000-inch (0.26 mm) STSGs were applied. On day 16 after admission, the Integra silicone sheet was removed, and 1:3 meshed 10/1000-inch (0.26 mm) STSGs were applied. Epithelialization had occurred in most parts of the burn; however, the graft failed in the proximal one-third portion of the left thigh, resulting in an ulcer. With topical treatment with a silver-impregnated hydrofiber dressing (Aquacel^®^ Ag BURN: ConvaTec, Princeton, NJ, USA) for 6 days, petroleum gel (PROPETO^®^; Maruishi Pharmaceutical Co., Tokyo, Japan) for 6 days, a hydrocortisone-bacterial culture suspension mixture ointment (Eksalb, Maruho, Osaka, Japan) for 3 days, and silicone-faced wound dressing mesh (SI-mesh; ALCARE, Tokyo, Japan) for 3 days, the ulcer was covered with healthy granulation tissue (Fig. 1: day 36).

On day 37 after admission, an operation was performed. The ulcer surface was debrided, and an 11/1000-inch (0.28 mm) skin graft was taken from the ipsilateral thigh and meshed, using a 1:1.5 ratio. A slightly larger skin graft was taken, and the excess skin graft was immediately regrafted to the donor site. The regrafted skin was meshed using a 1:1.5 ratio, the same as the recipient site. NPWT (INFOV.A.C.™ Therapy System, KCI, San Antonio, TX, USA) was then applied to both the donor and recipient sites with a continuous negative pressure of 125 mmHg. A silicone-faced wound dressing mesh (SI-mesh) was inserted between the skin graft and polyurethane foam dressing (V.A.C. GranuFoam Dressing, KCI, San Antonio, TX, USA) to avoid damage to the skin graft (Fig. 1: day 37). On days 37 and 38 after admission, 1 g of cefazolin was administered intravenously every 8 hours as perioperative prophylaxis.

**Fig. 2:**
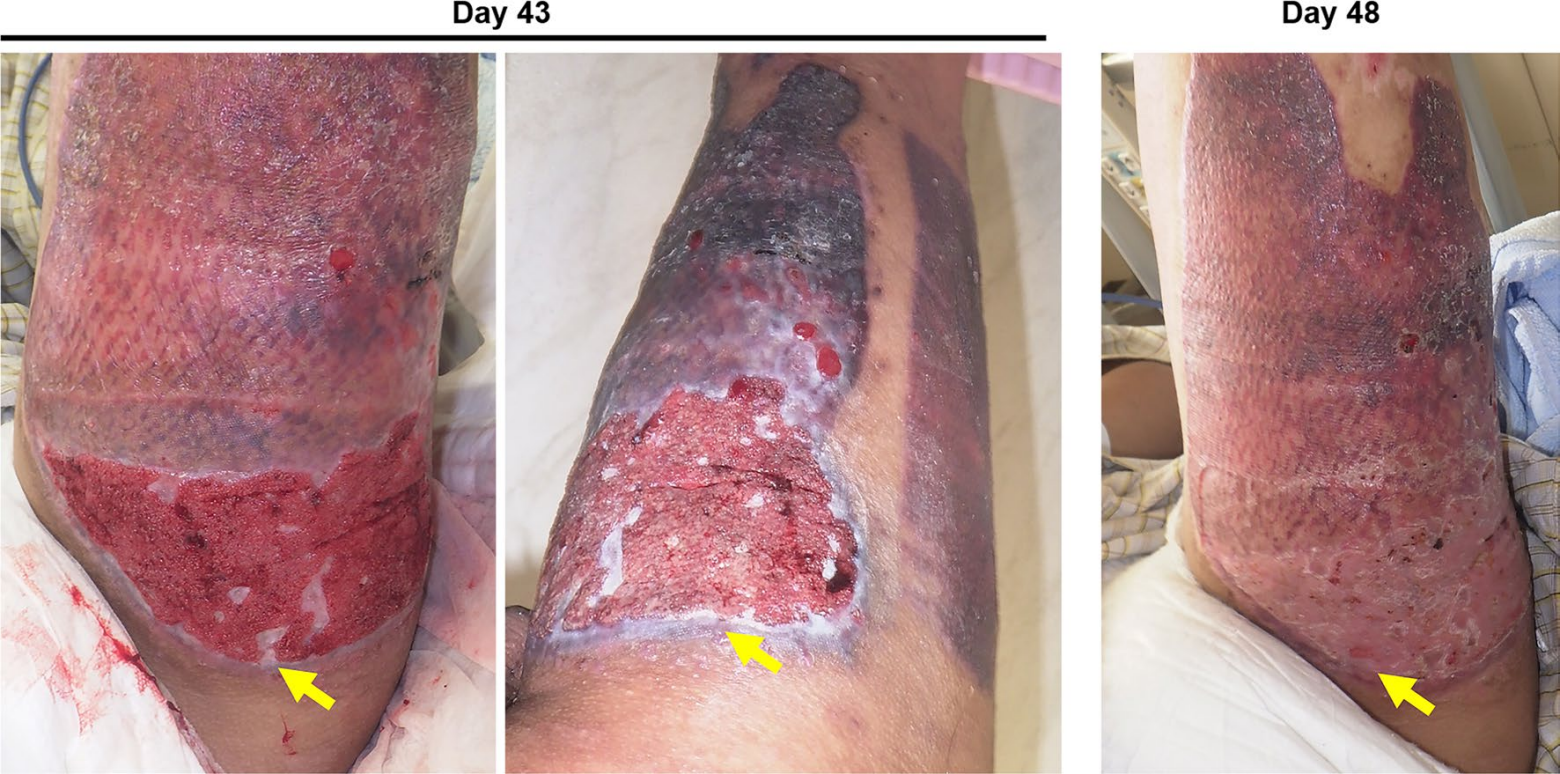
Postoperative state of the left thigh. Day 43: (Left) Immediately after NPWT was discontinued: the recipient site was covered with granulation tissue (arrow). (Right) Small islands of epithelialization were visible under the granulation tissue after the irrigation (arrow). Day 48: Epithelialization of the recipient site was almost completed (arrow)

On day 43 after admission, the polyurethane foam dressing was removed from both the donor and recipient sites. Although the regrafted skin at the donor site was taken completely, the skin graft was not found at the recipient site, and the entire area was covered with granulation tissue (Fig. 2: day 43, left). No granulation tissue overgrowth was observed at the donor site. The staples that had held the grafts in place were embedded in the granulation tissue and were removed. On the same day, the wound was cleaned under running tap water, and small islands of epithelialization were visible under the granulation tissue. NPWT was ceased, and topical treatment with a hydrocortisone-bacterial culture suspension mixture ointment (Eksalb) was applied (Fig. 2: day 43, right). Rapid epithelialization of the ulcer was observed as the granulation tissue regressed, and epithelialization of both the donor and recipient sites was almost completed on day 48 after admission (5 days after NPWT cessation) (Fig. 2: day 48). No significant change was observed between pre- and postoperative laboratory findings (Table 1). In addition, no significant findings were observed in urinalysis on day 17 (Table 2), and no further urinalysis was performed during hospitalization because no specific symptoms appeared. The patient was discharged on day 52 after admission and was referred to the local plastic surgery clinic for follow-up, and had recovered uneventfully at 6 months after surgery. The hand was healed without complications.

**Table 1:** Pre- and postoperative laboratory findings

	Preoperative (day 36)	Postoperative (day 51)	Unit	Reference range
White blood cells	7.18	7.00	10^3^/μL	3.3−8.6
Hemoglobin	10.4	10.6	g/dL	13.7−16.8
Hematocrit	32.8	34.3	%	40.7−50.1
Platelets	361	368	10^3^/μL	158−348
Total protein	6.7	6.6	g/dL	6.6−8.1
Albumin	3.9	3.9	g/dL	4.1−5.1
Creatine kinase	29	38	U/L	59−248
Aspartate aminotransferase	13	10	U/L	13−30
Alanine aminotransferase	14	7	U/L	10−42
Lactate dehydrogenase	143	126	U/L	124−222
Alkaline phosphatase	291	208	U/L	106−322
γ-Glutamyl transpeptidase	175	47	U/L	13−64
Amylase	42	43	U/L	44−132
Total bilirubin	0.4	0.4	mg/dL	0.4−1.5
Creatinine	0.64	0.72	mg/dL	0.65−1.07
Blood urea nitrogen	6.7	4.5	mg/dL	8.0−20.0
Uric acid	7.2	5	mg/dL	3.7−7.8
Sodium	137	141	mmol/L	138−145
Potassium	4.3	4.3	mmol/L	3.6−4.8
Chloride	102	107	mmol/L	101−108
Magnesium	2.4	2.2	mg/dL	1.8−2.4
Calcium	9.1	9.3	mg/dL	8.8−10.1
Inorganic phosphate	4.7	5.1	mg/dL	2.7−4.6
Zinc	115	Not measured	μg/dL	66−118
Copper	109	Not measured	μg/dL	68−128
C-reactive protein	1.42	0.52	mg/dL	0−0.14

**Table 2:** Preoperative urinalysis results

	Preoperative (day 17)	Reference range
Dipstick urinalysis		
Color	Pale yellow	–
Clarity	Clear	–
pH	7.5	–
Specific gravity	1.010	–
Glucose	Negative	Negative
Blood	Negative	Negative
Ketones	Negative	Negative
Protein	Negative	Negative
Urobilinogen	< 1.0 mg/dL	< 1.0 mg/dL
Bilirubin	Negative	Negative
Leukocyte esterase	Negative	Negative
Nitrite	Negative	Negative

## Discussion and conclusions

In this case, the meshed STSG was not found on the wound surface and was suspected to have failed. On the day of NPWT cessation, the granulation tissue had firmly covered the wound surface and could not be removed. Conservative treatment with ointment reduced the size of the granulation tissue, and the epithelized areas expanded rapidly. These findings suggest that the hypergranulation tissue had overgrown and covered the meshed skin graft. Although protrusion of the hypergranulation tissue through the mesh interstices of the skin graft is not uncommon when NPWT is used for the fixation of meshed skin grafts [1], to the best of our knowledge, there have been no reports that the meshed STSG was fully covered by the granulation tissue induced by NPWT.

**Fig. 3:**
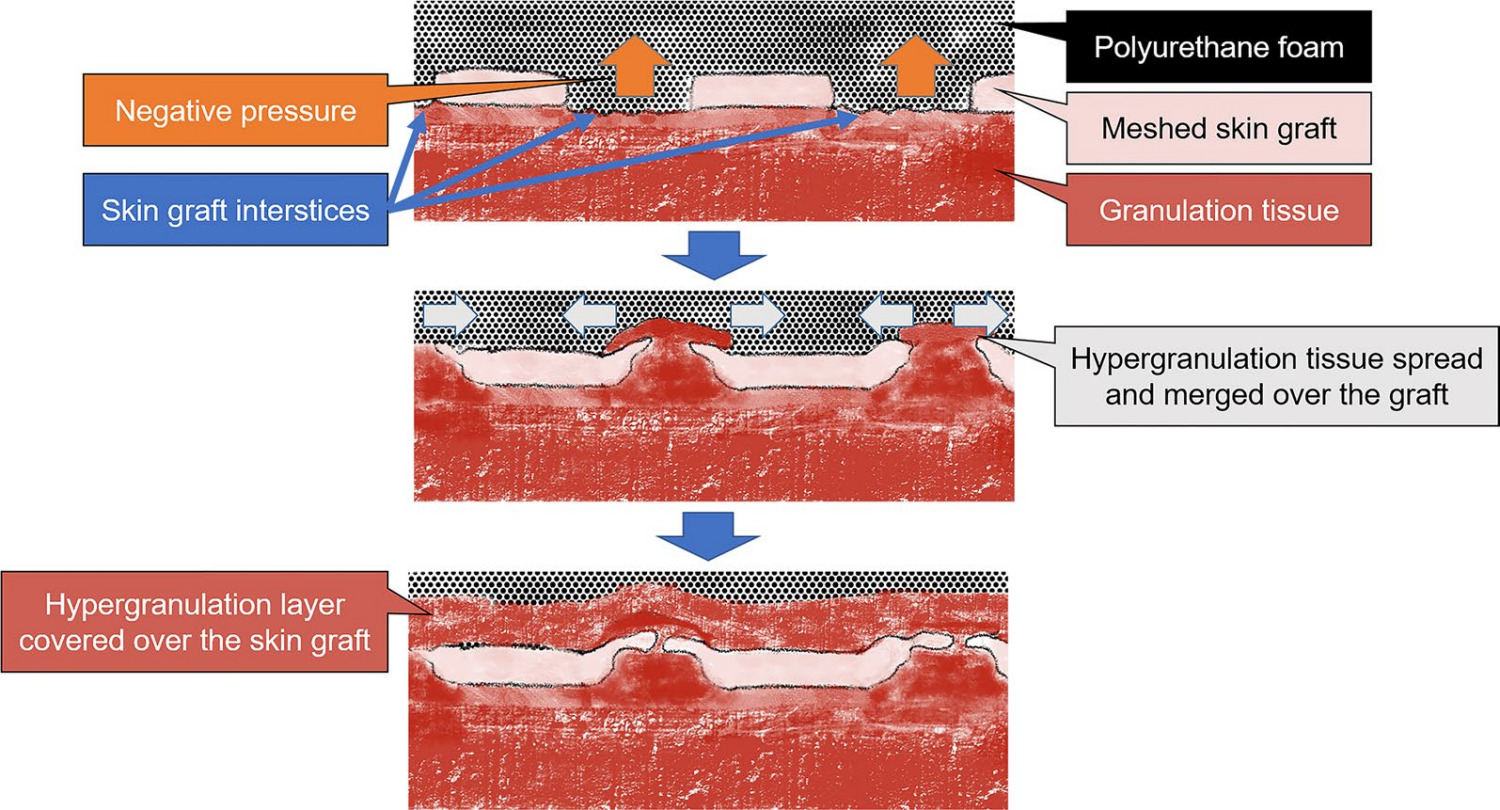
Possible mechanism underlying the phenomenon involving covering of the skin graft with granulation tissue. Negative pressure was applied to the healthy granulation tissue through the skin graft interstices, which resulted in the rapid growth of hypergranulation tissue and the formation of a hypergranulation layer

The possible reason that the skin graft was covered by the hypergranulation tissue is that the rate of granulation stimulated by NPWT was faster than the rate of closure of the meshed STSG interstices (Fig. 3). The granulation tissue might protrude through the mesh interstices, and the overgrown granulation tissue might have spread and merged over the graft. The negative pressure applied to the healthy granulation tissue might have induced unusually rapid hypergranulation tissue formation. Once NPWT was discontinued, the hypergranulation tissue resolved quickly, and the interstices closed. In a previous report, an 87-year-old man with post-burn Achilles tendon exposure resulting from a full-thickness burn underwent NPWT, and no granulation tissue was observed after 10 days of NPWT [5]. The authors made two longitudinal slits penetrating the exposed Achilles tendon to deliberately induce blood flow from the ventral side to the dorsal surface. After 28 days of continuous NPWT, the authors confirmed that the Achilles tendon was completely covered with well-vascularized granulation tissue. They pointed out that the formation of the granulation tissue was induced by the longitudinal slits penetrating the Achilles tendon, which induced the blood flow to the dorsal surface from the ventral surface. This phenomenon is similar to our case. In our case, the skin graft interstices might have worked in a similar way as the longitudinal slits. This would explain the mechanism of hypergranulation tissue merging over the meshed STSG. Another possible reason for rapid granulation may have been that the negative pressure was set slightly higher for skin fixation, and the duration of the procedure was longer, relative to the previous report [1], although skin fixation using NPWT with a longer duration or the same negative pressure has been reported [2, 6]. In the search for the optimal negative pressure for NPWT for STSG fixation, it was observed that pressure settings as low as 50 mmHg may be tolerated without compromise to epithelialization [6]. No significant complications were encountered in that study with a maximum negative pressure setting of 125 mmHg. This indicates that a high negative pressure (that is, 125 mmHg) is not sufficient to induce hypergranulation over the meshed STSG. In this situation, a polyvinyl alcohol foam dressing might be more favorable than a polyurethane foam dressing for NPWT because its increased density and smaller pores may restrict the ingrowth of granulation tissue [4]. No histological examination was performed in this case, and the final cause was not determined.

In this case, on the day of NPWT cessation, the hypergranulation tissue was raised and covered almost the entire wound surface. It was difficult to scrape the granulation tissue, and there was no evidence of the skin graft under the granulation tissue at that time. Debridement in this condition is highly invasive and involves a high risk of skin graft damage. Rapid epithelialization was completed 5 days after NPWT cessation without any surgical interventions. If immediate debridement and skin grafting had been performed in this case, it would have resulted in the loss of the initially taken skin graft, which would have caused an unnecessary complication for this patient. Once surgeons determine that the appearance of the wound is due to the hypergranulation tissue over the integrated grafts, topical steroid dressings would be a good treatment choice [7]; a hydrocortisone-bacterial culture suspension mixture ointment was used in this case. Although this is rare, it is a reversible condition that should be recognized when using NPWT for STSG fixation.

In conclusion, hypergranulation over a meshed STSG is a rare complication when the graft is fixed by NPWT. It is important to note that immediate debridement should be avoided, and conservative treatment should be initiated because the graft may be integrated under the granulation tissue.

## Data Availability

The datasets obtained and analyzed in the current study are available from the corresponding author upon reasonable request.

## Abbreviations

NPWT: Negative-pressure wound therapy
STSG: Split-thickness skin graft
